# The influence of perceived teacher support on online English learning engagement among Chinese university students: a cross-sectional study on the mediating effects of self-regulation

**DOI:** 10.3389/fpsyg.2024.1246958

**Published:** 2024-02-13

**Authors:** Danting Yin, Lin Luo

**Affiliations:** ^1^Department of General Education, Sichuan Vocational College of Cultural Industries, Chengdu, Sichuan, China; ^2^School of Physical Education, Guizhou Normal University, Guiyang, Guizhou, China

**Keywords:** online learning engagement, perceived teacher support, self-regulation in learning, English language education, Chinese university students, educational psychology, distance learning

## Abstract

**Objectives:**

This study examines the association between perceived teacher support and self-regulation in learning, and their combined relationship with online English learning engagement among university students in China. The objective is to uncover the underlying mechanisms of this relationship, with a particular focus on the role of self-regulation in learning as a mediator.

**Methods:**

The study involved 1,361 university students from Southwest China, predominantly female (73.84%) with an average age of 18.94 years (SD = 1.07). Refined measurement tools were employed to assess perceived teacher support, online English learning engagement, and self-regulation in learning.

**Results:**

The findings indicate that components of self-regulation, such as goal setting, environmental structuring, and time management, act as full mediators in the relationship between perceived teacher support and online English learning engagement.

**Conclusion:**

This research underscores the importance of self-regulation in learning in linking perceived teacher support with online English learning engagement. The insights gained are crucial for enhancing teaching strategies in online English language education.

## 1 Introduction

In the contemporary academic landscape, university students, often termed “digital natives” (Murray and Pérez, 2014), have grown alongside the rapid evolution of digital technologies. Their inherent digital literacy, augmented by widespread digital applications, has significantly influenced the higher education sector, fostering educational reforms and sustainable development (Fonseca et al., 2014). The global COVID-19 pandemic catalyzed a shift toward online platforms, with over 1.18 billion university students engaging in 7.1 million online courses during China’s lockdown period alone (Huang et al., 2020). This digital transition in education has yielded mixed outcomes: while some studies report increased student interest and engagement in learning (Shi and Yu, 2016; Tang and Carr-Chellman, 2016), challenges such as high dropout rates persist (Wu and Bai, 2018).

Online language education, in particular, has garnered attention for its potential to alleviate learner anxiety and enrich the learning experience (Choi and Chung, 2021). However, the integration of essential nonverbal communication skills in online settings remains challenging, potentially diminishing teaching effectiveness and learning outcomes (Sands, 2002). Online language learners are compelled to harness a range of behavioral, cognitive, and affective skills to navigate the complexities of digital learning environments (Zheng et al., 2018). The ecological systems theory posits that student engagement emerges from multifaceted environmental interactions (Scott, 1980), with the symbiotic efforts of educators and students playing a pivotal role in enhancing engagement (Vollet et al., 2017). Research has explored the nuances of teacher support in relation to students’ cognitive, affective, and social engagement (Luan et al., 2023), yet the specificities of its impact on online learning engagement necessitate further investigation.

Self-regulated learning strategies in online contexts have been identified as pivotal predictors of academic success (Wang et al., 2013). Online learning, devoid of traditional time and space constraints, demands a heightened level of independent student self-management and regulation (Allen and Seaman, 2016). Recent findings have underscored a significant correlation between online self-regulated learning strategies and student engagement (Harrad et al., 2023), emphasizing the need for language educators to cultivate positive learning environments that bolster student interest and motivation (Choi and Chung, 2021).

Guided by the ecological systems theory of education, this study seeks to elucidate the influence of Perceived Teacher Support and online self-regulated learning strategies on the online English learning engagement of students. Furthermore, it aims to clarify the mediating role of self-regulation in learning within this relationship. The anticipated outcomes of this research endeavor are twofold: to deepen our understanding of student engagement in online learning contexts and to offer strategic insights for optimizing online English language education.

### 1.1 Perceived teacher support

Kasap (2021) underscores the significance of creating a learning environment that addresses students’ fundamental psychological needs for autonomy, competence, and relatedness, thereby fostering self-regulated learning (SRL) and indirectly enhancing academic achievement. In such environments, students’ subjective perceptions of support and assistance from teachers, defined as perceived teacher support, are vital. Teacher support encompasses emotional, cognitive, and behavioral dimensions, including encouragement, feedback, guidance, attention, and understanding. From the educator’s standpoint, providing instructional support that aligns with students’ basic SRL needs is critical. Teacher support has evolved to encompass two main types: academic and emotional support (Liu et al., 2018). Academic support relates to students’ perceptions of their teachers’ effort and instructional effectiveness, while emotional support pertains to the degree of care, respect, and emotional assistance experienced by learners (Buckman et al., 1985). Despite their relative maturity, university students, especially in language learning contexts, continue to require both emotional and instrumental support (Kormos and Csizer, 2014). This study, therefore, concentrates on examining the impact of both emotional and instrumental support provided by foreign language teachers in online English course settings.

### 1.2 Online English learning engagement

Online student engagement is characterized by the time and effort students invest in online learning processes (Ma et al., 2015). It is a multidimensional construct, often conceptualized as comprising behavioral, affective, and cognitive dimensions (Blumenfeld et al., 2004; Jung and Lee, 2018). In the context of online learning, behavioral engagement involves activities such as question-asking and participation in discussions, while affective engagement pertains to positive emotional experiences with teachers, peers, and the course content. Cognitive engagement refers to the mental effort exerted by students to acquire complex knowledge or develop specific skills (Ding et al., 2017; Jung and Lee, 2018). The National Survey of Student Engagement (NSSE) has developed a comprehensive survey encompassing dimensions like academic challenges, supportive campus environments, enriching educational experiences, and faculty-student interaction to gauge online student engagement (Blumenfeld et al., 2004). In line with social constructivism, Dixson (2015) developed an involvement scale to explore the relationship between students’ nonverbal behaviors and online course engagement, emphasizing the multidimensional nature of student engagement (Dixson, 2015). This study, therefore, focuses on dissecting the dimensions of skills, affect, engagement, and performance in the realm of students’ online learning engagement.

### 1.3 Self-regulation in learning

A key challenge in online learning is the effective self-regulation of metacognition, motivation, and behavior by students. Self-regulation in learning strategies have a proven association with academic performance (De Groot and Pintrich, 1990) and are deemed essential for lifelong learning (Dehmel, 2006). The proliferation of digital information and online learning modalities in recent decades has shifted the focus of self-regulation research from traditional classrooms to digital environments (Winne, 2005). This transition presents a challenge for educators, as online learning necessitates greater self-direction rather than direct guidance (Artino and Stephens, 2009). Research indicates that self-regulation skills are particularly beneficial for online learning environments (Hill and Song, 2009), with significant implications in MOOCs (Hood et al., 2015) and various case studies (Alhazbi and Hasan, 2021). Empirical studies on self-directed online learning have increased, leading scholars to develop a six-factor scale to measure self-regulation in learning in online contexts, including goal setting, time management, environmental structuring, help-seeking, task strategies, and self-evaluation (Lai and Gu, 2011). The efficacy of online learning, particularly in English language education, is significantly enhanced by robust self-regulation skills (Kormos and Kiddle, 2013; Zheng et al., 2018). Furthermore, research has illuminated the relationship between teacher instruction and student self-regulation in learning (Agran et al., 2001), suggesting a potential link between perceived teacher support and self-regulation in online English language learning.

### 1.4 The relationship between perceived teacher support, self-regulation in learning, and online English learning engagement

**Figure 1:**
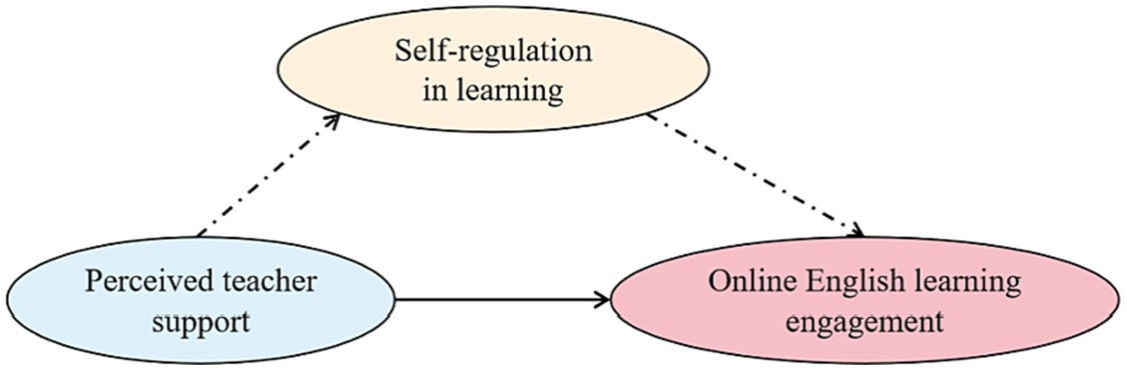
Hypothesized model.

Since the inception of computer-based learning, there has been a notable improvement in classroom interaction and learning engagement (Fried, 2008). Studies such as those by Gosper et al. (2011) reveal that the use of social media by students can enhance academic performance and adaptability, contributing to learning engagement. However, the transition from traditional to online learning environments presents distinct challenges (Wester et al., 2021). To foster sustained online language learning, educators must cultivate positive environments that stimulate student interest and motivation (Choi and Chung, 2021). Self-regulation has been linked to learning engagement, a relationship that persists in online settings (Winne, 2005). Consequently, it is hypothesized that a significant positive relationship exists between students’ perceived teacher support, self-regulation in online English learning, and learning engagement. Furthermore, the advent of big data has improved online learning outcomes. Engaged students exhibit heightened self-regulation, attentiveness, participation, and effort in learning (Blumenfeld et al., 2004). Students’ perceptions of teacher support, such as timely feedback, correlate positively with engagement (Luan et al., 2023). Given the diversity of learning environments, students must adapt their self-regulation skills to optimize learning outcomes, a crucial factor for their sustainable development in academic and career trajectories. To validate the relationships between teacher support, self-regulation in online learning, and English learning engagement, this study proposes the following research model (see Figure 1) and posits the subsequent research hypotheses:

*H*1: Perceived teacher support significantly and positively impacts online English learning engagement.

*H*2: Perceived teacher support significantly and positively impacts self-regulation in learning.

*H*3: Self-regulation in learning significantly and positively impacts online English learning engagement.

*H*4: Perceived teacher support indirectly influences online English learning engagement through self-regulation in learning.

## 2 Methods

In this cross-sectional study, data collection occurred from March to April 2023 via the “Maike” platform. The survey’s QR code was disseminated through the online social platform WeChat, specifically targeting university students. Participants, after providing informed consent, voluntarily completed the questionnaire. The study’s inclusion criteria were: (i) an age range of 18 to 29 years, (ii) proficiency in using digital media tools, and (iii) enrollment in courses for English as a second language. Responses failing to meet the age requirements or providing illogical answers (such as reporting a non-English language as their language of study) were deemed invalid. This research was conducted with the approval of the Ethics Committee of the College of Physical Education at Guizhou Normal University, China (GZNUPEI20220524), adhering to the standards outlined in the latest iteration of the Helsinki Declaration. Initially, 1,421 volunteers participated in the survey. After rigorous screening, data from 1,361 university students (mean age 18.94 ± 1.07 years) across various provinces were included for analysis. Female participants constituted a significant majority of the sample, accounting for 73.84%. Detailed demographic characteristics of the participants are delineated in Table 1.

**Table 1:** Descriptive statistic and Cronbach’s alpha values of primary outcomes (*n* = 1,361).

Variables	Mean	SD	Cronbach’s alpha
Perceived teacher support	46.669	8.119	0.964
Emotional support (ES)	23.325	4.139	0.925
Instrumental support(IS)	23.345	4.284	0.955
Self-regulation in learning	72.253	14.672	0.967
Goal setting(GS)	15.655	3.741	0.917
Environmental structuring(EnS)	13.163	2.875	0.888
Task strategies(TS)	12.31	2.816	0.849
Time management(TM)	9.424	2.233	0.891
Seeking help(SH)	12.444	2.772	0.853
Self-evaluation(SE)	9.256	2.257	0.898
Online English learning engagement(OELE)	56.587	11.374	0.957
Skills	15.934	3.391	0.896
Emotion	16.206	3.429	0.909
Participation	18.73	4.186	0.894
Performance	5.717	1.66	0.912

### 2.1 Measurements

#### 2.1.1 Demographic information

Collecting demographic information, including gender (male & female), age, residential area (rural, urban, city), grade level (freshman, sophomore, junior, senior), and self-rated English proficiency (very poor, poor, normal, good, excellent).

#### 2.1.2 Perceived teacher support

The perceived teacher support was measured using the Student Perceived Teacher Support Scale developed by Federici and Skaalvik (2014). This scale comprises two dimensions: Emotional Support and Instrumental Support. The process of translating and revising the scale was similar to that of the English Class Performance Anxiety questionnaire. Prior to the formal study, the Chinese version of the scale had been validated. In this research, the validated Student Perceived Teacher Support Scale was used to assess the level of teacher support as perceived by students. The scale consists of 12 items, with 6 items each for the dimensions of Emotional Support and Instrumental Support. These items are rated on a Likert 5-point scale, ranging from 1 (“strongly disagree”) to 5 (“strongly agree”). The total score is obtained by summing all responses, with higher scores indicating higher levels of perceived teacher support. In this study, the reliability and validity of the Chinese version of the Student Perceived Teacher Support Scale were acceptable, with a Cronbach’s alpha of 0.964.

#### 2.1.3 Online English learning engagement

To assess the level of online English learning engagement, we utilized the Online Student Engagement (OSE) Scale developed by Dixson et al. (2017), which consists of 19 items across four dimensions: skills (5 items), emotion (5 items), participation (6 items), and performance (3 items). The revision process of this scale followed the same procedure as the Perceived Teacher Support scale. Prior to the formal study, the Chinese version of the scale had already been validated. Following the revision, the Chinese version of the Online English Learning Engagement scale retained 2 items for the performance dimension, while keeping the rest of the dimensions unchanged, resulting in a total of 18 items. In this study, the validated Online English Learning Engagement scale was employed to measure students’ level of engagement in online English learning. A 5-point Likert scale ranging from 1 (“strongly disagree”) to 5 (“strongly agree”) was used to rate each item. The scores of all responses were summed to obtain a total score. Higher total scores indicate a higher level of online English learning engagement. In this study, the reliability and validity of the Chinese version of the online English learning engagement scale were acceptable, as indicated by a Cronbach’s alpha coefficient of 0.957.

#### 2.1.4 Self-regulation in learning

The Self-Regulated Learning (SRL) Scale developed by Barnard-Brak et al. (2010) was used to measure self-regulated learning. The scale consists of 24 items, divided into six dimensions: Goal setting (5 items), environmental structuring (4 items), task strategies (4 items), time management (3 items), seeking help (4 items), and self-evaluation (4 items). The translation and revision process of the scale followed the same procedure as the Perceived Teacher Support scale. Prior to the formal study, the Chinese version of the scale had already been validated. Following the revision, the Chinese version of the Self-Regulated Learning scale retained 3 items for the self-evaluation dimension, while keeping the rest of the dimensions unchanged, resulting in a total of 23 items. In order to maintain consistency with the observed outcome variable, English language learning was added as a qualifier when measuring self-regulated learning in this study. The validated Self-Regulated Learning scale was used to assess students’ ability in self-regulated learning. A 5-point Likert scale ranging from 1 (“strongly disagree”) to 5 (“strongly agree”) was used to rate each item. The scores of all responses were summed to obtain a total score. A higher total score indicates a stronger ability in self-regulated learning. In this study, the reliability and validity of the Chinese version of the Self-Regulated Learning scale were acceptable, as indicated by a Cronbach’s alpha coefficient of 0.967.

### 2.2 Statistical analysis

In this study, we used SPSS 26.0 (Armonk, NY, United States) to calculate the sample distribution, means, standard deviations, and Pearson correlations among the variables in our theoretical model. Normality checks using the Doornik-Hansen test were conducted on the variables of interest, including age, online English learning engagement, perceived teacher support, and self-regulation in learning. The following criteria were used to assess the correlation coefficients: no correlation: ≤ 0.19; low correlation: 0.20–0.39; moderate correlation: 0.40–0.59; moderate to high correlation: 0.60–0.79; high correlation: ≥ 0.80 (Cohen and Soffer, 2019). Additionally, Cronbach’s alpha was calculated to estimate the internal consistency of specific scales, where a Cronbach’s alpha >0.7 indicates high internal consistency reliability (Sawyer et al., 2014). Bootstrapping tests with 5,000 samples and a 95% confidence interval were used to determine the total effects, indirect effects, and direct effects among the variables (Lamp et al., 2021).

## 3 Results

### 3.1 Descriptive statistics and internal consistency reliability

Table 1 presents the means, standard deviations, and Cronbach’s alpha values for the continuous variables. The primary observed results and their corresponding scales demonstrate acceptable reliability (Cronbach’s alpha coefficients = 0.849 to 0.967).

### 3.2 Correlation analysis

Table 2 displays the correlation results between variables. Perceived teacher support showed positive correlations with self-regulation in learning, goal setting, environmental structuring, task strategies, time management, self-evaluation, online English learning engagement (*r* = 0.340, 0.324, 0.366, 0.282, 0.260, 0.275, 0.261, 0.254, *p* < 0.01). Online English learning engagement also exhibited positive correlations with self-regulation in learning, goal setting, environmental structuring, task strategies, time management, and self-evaluation (*r* = 0.801, 0.667, 0.653, 0.696, 0.712, 0.743, 0.781, *p* < 0.01) between each pair of variables.

**Table 2:** Correlations for variables (*n* = 1,361).

Variables	PTS	ES	IS	GS	EnS	TS	TM	SH	SE	OELE
**Perceived teacher support (PTS)**	1									
Emotional support (ES)	0.963**	1								
Instrumental support(IS)	0.965**	0.858**	1							
Self-regulation in learning	0.340**	0.299**	0.356**	1						
Goal setting(GS)	0.324**	0.283**	0.340**	0.877**	1					
Environmental structuring(EnS)	0.366**	0.335**	0.370**	0.870**	0.762**	1				
Task strategies(TS)	0.282**	0.243**	0.299**	0.901**	0.724**	0.745**	1			
Time management(TM)	0.260**	0.223**	0.277**	0.899**	0.732**	0.727**	0.819**	1		
Seeking help(SH)	0.275**	0.242**	0.288**	0.876**	0.652**	0.674**	0.758**	0.780**	1	
Self-evaluation(SE)	0.261**	0.226**	0.277**	0.848**	0.644**	0.644**	0.722**	0.733**	0.812**	1
**Online English learning engagement (OELE)**	0.254**	0.222**	0.267**	0.801**	0.667**	0.653**	0.696**	0.712**	0.743**	0.781**

*p < 0.05; **p < 0.01; The bold text is the total amount table.

### 3.3 Linear regression analysis

The research findings indicate (Table 3) that residence, English proficiency, self-evaluation, seeking help, time management, goal setting, and environmental structuring have a significant positive impact on online English learning engagement. However, gender, age, grade, instrumental support, emotional support, and task strategies do not have an impact on online English learning engagement.

**Table 3:** Linear regression analysis results (*n* = 1,361).

	Standardized coefficient	*t*	*p*	95% CI
Gender	0	0.017	0.986	−0.777 ~ 0.791
Age	−0.016	−0.973	0.331	−0.508 ~ 0.171
Residence	0.041	2.638	0.008	0.153 ~ 1.036
English proficiency	0.091	5.667	<0.001	0.781 ~ 1.607
Grade	0.027	1.648	0.100	−0.153 ~ 1.774
Instrumental support(IS)	−0.002	−0.081	0.935	−0.164 ~ 0.151
Emotional support (ES)	0.005	0.169	0.866	−0.146 ~ 0.173
Self-evaluation(SE)	0.409	14.827	<0.001	1.788 ~ 2.333
Seeking help(SH)	0.167	5.524	<0.001	0.441 ~ 0.926
Time management(TM)	0.075	2.417	0.016	0.072 ~ 0.690
Task strategies(TS)	0.049	1.609	0.108	−0.043 ~ 0.435
Goal setting(GS)	0.127	4.838	<0.001	0.230 ~ 0.544
Environmental structuring(EnS)	0.066	2.454	0.014	0.053 ~ 0.470

### 3.4 Mediation analysis

#### 3.4.1 Common method bias test

This study employed the Harman single-factor test to examine common method bias. All items were analyzed using principal component analysis in SPSS. The results showed that the first eigenvalue accounted for only 32.67% of the variance, which is less than 40%, indicating no issues of common method variance (Kock, 2015). Additionally, we assumed a single common factor and conducted a confirmatory factor analysis with all items as observed variables. The results of the confirmatory factor analysis indicated that the model fit indices (χ^2^/df = 18.026, GFI = 0.293, IFI = 0.439, NFI = 0.441, RMSEA = 0.112) were not ideal. This suggests that there is no significant common method bias among the variables. Therefore, in further statistical analysis, no variables were excluded or merged.

#### 3.4.2 Hypothesis path testing

**Figure 2:**
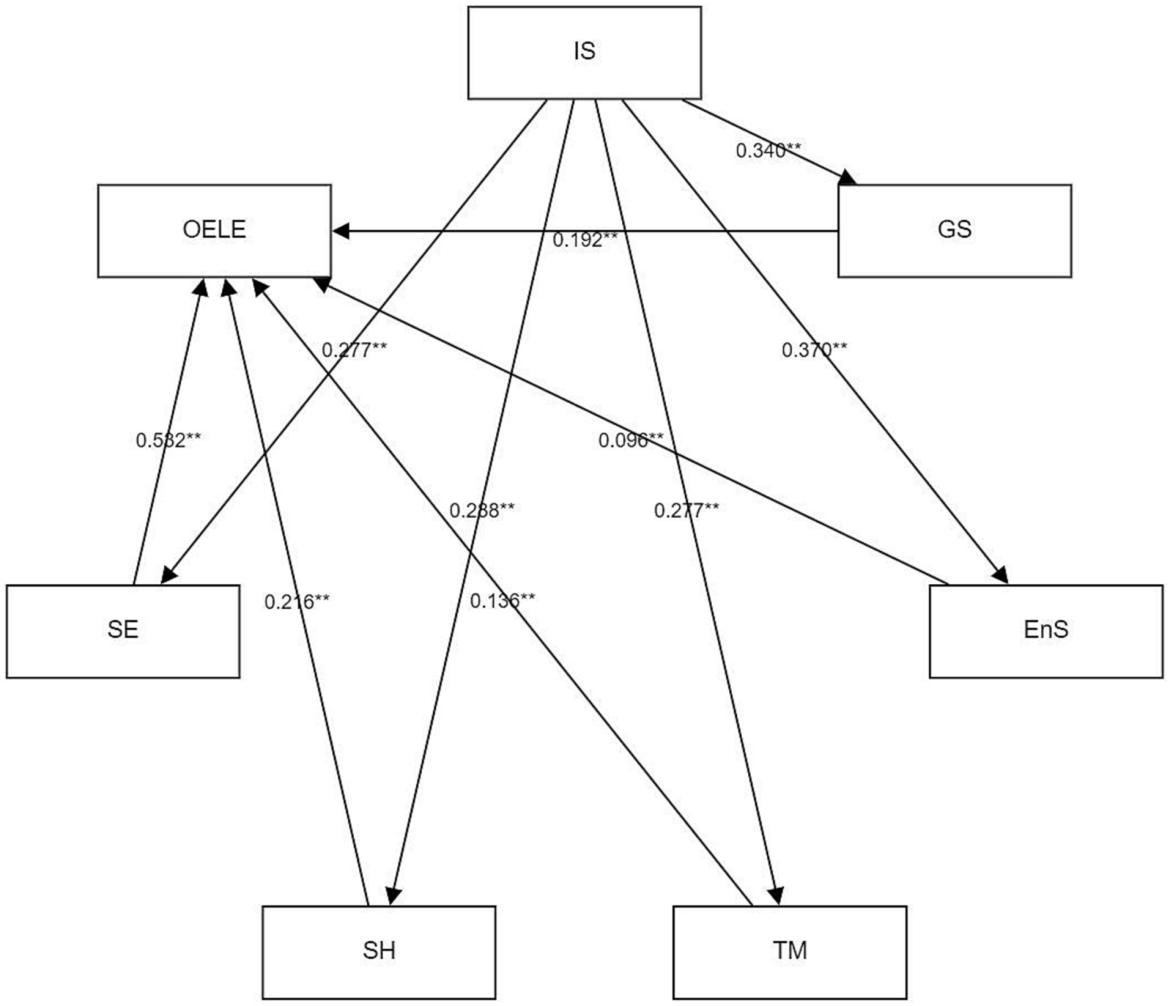
Path testing results. S, emotional support; IS, instrumental support; GS, goal setting; EnS, environmental structuring; TS, task strategies; TM, time management; SH, seeking help; SE, self-evaluation; OELE, online English learning engagement.

Integrating Perceived Teacher Support and Self-regulation in learning into the path analysis model represents a significant enhancement to our analytical framework. These two factors, Perceived Teacher Support and Self-regulation, play pivotal roles in influencing Online English learning engagement directly. Figure 2 provides a visual representation of the path diagram, shedding light on the intricate relationships within our model. It illustrates how instrumental support, a key component, indirectly impacts college students’ online English learning engagement. This indirect influence is mediated through a series of crucial self-regulation processes, including self-evaluation, seeking help, time management, goal setting, and environmental structuring. The fit indices for the path model are as follows (GFI = 0.903, CFI = 0.903, NFI = 0.913, RMSEA = 0.09).

## 4 Discussion

This study embarked on an exploration of student behaviors in online English learning environments, concentrating on the interplay among Perceived Teacher Support, Online Learning Engagement, and Self-regulation. Through mediation analysis, the intricate relationships between these variables were systematically examined.

The investigation confirmed a significant positive correlation between Perceived Teacher Support and Online Learning Engagement among Chinese university students engaged in distance foreign language learning. This aligns with existing scholarly discourse, highlighting a robust connection between students’ perceptions of teacher support and their learning behaviors. Research by Luan et al. (2023) underscored increased engagement in online English learning among Chinese university students who experienced substantial teacher support. Similarly, Wang et al. (2017) observed that students who perceived care, respect, and autonomy from their educators demonstrated enhanced academic compliance and reduced disruptive behaviors. Hew (2016) emphasized the significance of teacher communication, enthusiasm, and resource provision in elevating students’ engagement levels in online learning environments. These findings suggest that teachers act as more than subject matter experts; they are mentors who significantly influence the learning journey (Czerkawski and Lyman, 2016). The cultural reverence for teacher authority within the Chinese context, as discussed by Zhang (2010), may offer additional insight into these results.

Moreover, a compelling positive correlation emerged between Online Learning Engagement and Self-regulation. This suggests that effective Self-regulation strategies can amplify focus and commitment in online English learning contexts. Consistent with the findings of Wang et al. (2022), Self-regulation was identified as a key predictor of learning engagement. Bergmann (2017) also highlighted the influential role of Self-regulation in determining learning outcomes in flipped classroom settings. Students exhibiting higher levels of Self-regulation are generally more actively engaged in learning activities (Bohlmann and Downer, 2016), a trend that extends to English writing tasks (Teng and Zhang, 2020).

The study revealed that Perceived Teacher Support indirectly influences Online Learning Engagement through Self-regulation processes like goal setting, environmental structuring, and time management. This highlights the vital role of Self-regulation strategies in mediating the relationship between Perceived Teacher Support and Online Learning Engagement. Notably, the impact of Affective Support versus Instrumental Support within Perceived Teacher Support on student engagement exhibited variability. Affective Support was found to have a minimal direct impact on active learning participation, whereas Instrumental Support significantly fostered Self-regulation and active engagement. Although Affective Support did not directly elevate Online Learning Engagement, it played a role in fostering various Self-regulation strategies.

While these findings offer valuable insights, their interpretation must be contextualized within the constraints of the study’s limitations. The cross-sectional research design employed here precludes the establishment of causal relationships and the observation of longitudinal trends. Additionally, the concentration on a student cohort from Southwest China potentially limits the generalizability of the results, prompting questions regarding their relevance to other national and cultural contexts. Notably, this study predominantly featured a female student sample, which may introduce biases or specific tendencies that might not be representative of a more gender-balanced or male-dominated sample. Such a demographic skew could influence the study’s findings, particularly in aspects related to engagement and perception of teacher support. Moreover, the potential impact of students’ English proficiency on Online Learning Engagement was not thoroughly examined. Given the multifaceted nature of factors influencing Online English Learning Engagement, further research is needed to corroborate these findings in diverse second language learner populations. Future studies should consider integrating additional variables like digital media usage characteristics (Wang et al., 2022) and peer social support (Luan et al., 2023), as well as exploring gender differences in learning engagement and perception of teacher support, to enhance the understanding of the dynamics between Perceived Teacher Support and student Online English Learning Engagement.

In conclusion, this study makes a substantial contribution to the theoretical understanding of online English learning by elucidating the roles of teacher support and self-regulation in student engagement. The findings enrich the existing body of knowledge in educational ecology, specifically highlighting the nuanced impacts of instrumental and affective teacher support in an online context. Theoretically, this research expands upon the dynamics of student engagement, offering a more detailed view of the interplay between external support systems and internal student strategies. From a practical standpoint, the insights gleaned from this study have significant implications for the design and implementation of online English learning programs. Educators and curriculum designers can utilize these findings to create more effective online learning environments, tailoring teacher support to foster greater student engagement and self-regulation. The differentiation between instrumental and affective support provides a clear direction for educators on how to balance these two aspects to maximize student engagement and learning outcomes.

## 5 Conclusion

The study’s findings highlight the pivotal role of learners’ self-regulation strategies in mediating the connection between teacher support and their engagement in the learning process. Significantly, it was observed that instrumental support plays a more substantial role than affective support in bolstering students’ engagement in online English learning. For future research endeavors, it would be insightful to extend this examination to varied cultural and educational settings, thereby evaluating the generalizability of these findings. Moreover, a focused exploration into how digital literacy interacts with self-regulation strategies in online learning across various academic disciplines promises to enrich our comprehension of what constitutes effective and engaging online education methodologies.

## Data availability statement

The raw data supporting the conclusions of this article will be made available by the authors, without undue reservation.

## Ethics statement

This study has been approved by the Ethics Committee of Guizhou Normal University (GZNUPEI20220524). Participants were assured that their personal identities would not be disclosed in subsequent research reports.

## Author contributions

DY participated in data collection, literature review, and manuscript writing. LL participated in study design, data analysis, and manuscript quality check. All authors contributed to the article and approved the submitted version.
